# Supplementary material for: Stress increases hepatic release of lipocalin 2 which contributes to anxiety-like behavior in mice
Source: Nat Commun. 2024 Apr 8;15:3034. doi: 10.1038/s41467-024-47266-9 (PMC11001612; doi:10.1038/s41467-024-47266-9)
Supplement: Supplementary file 1 — Supplementary Information [file 41467_2024_47266_MOESM1_ESM.pdf]

Supplemental Information

Stress increases hepatic release of lipocalin-2 which contributes to anxiety-like behaviour in mice

Figures S1-S11

Table S1-S2

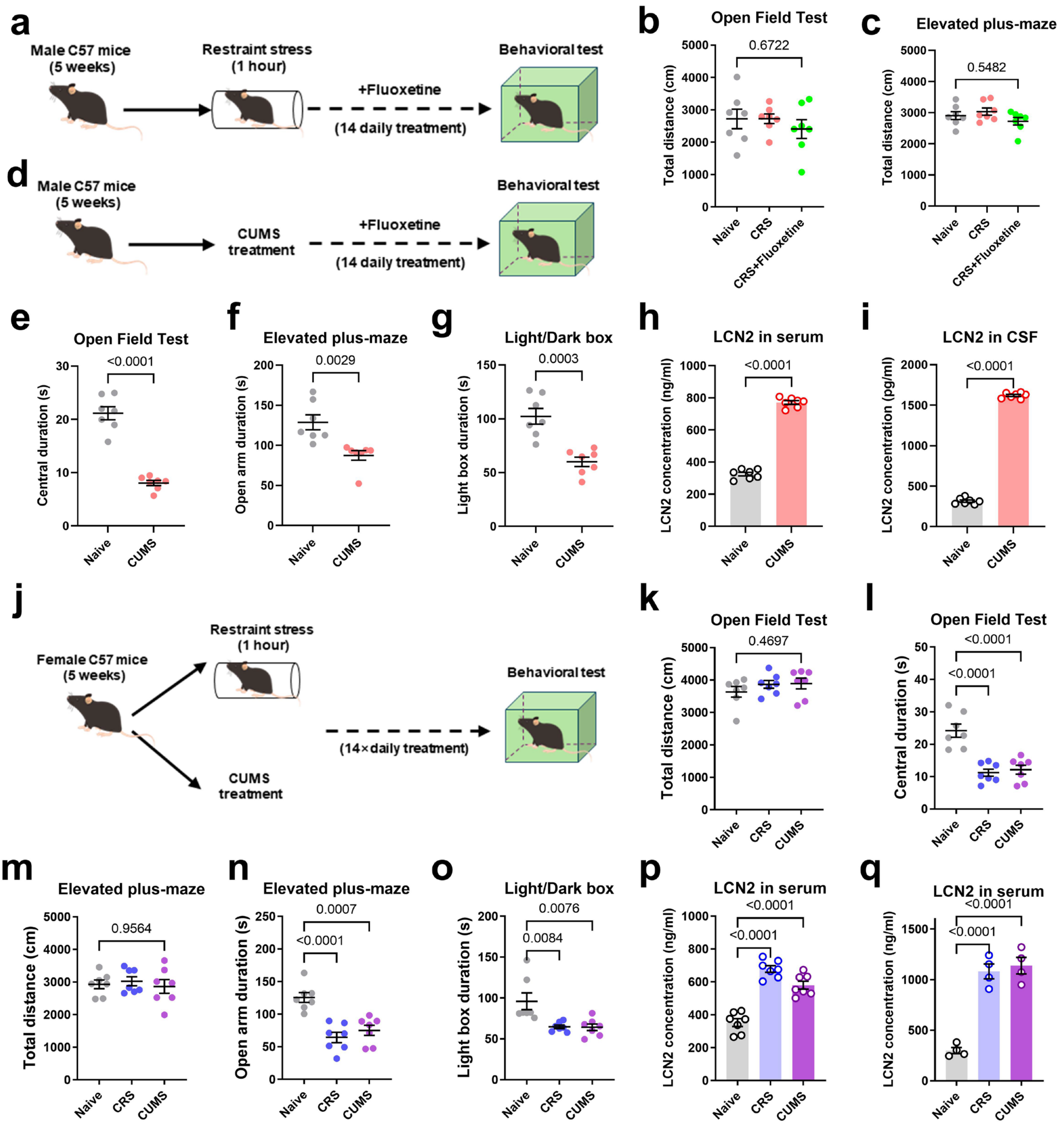

Figure S1. Peripheral LCN2 is correlated with anxiety-like behaviors under stress. (a) Time schedule of chronic restraint stress (CRS) paradigm. **(b)** No significant change of total distance in the open field. One-way ANOVA, $F(2,18)=0.4995$, $P=0.6722$. **(c)** No change of locomotor activity in the elevated plus-maze. $F(2,18)=1.648$, $P=0.5482$. **(d)** Time schedule of chronically unpredicted

mild stress (CUMS) paradigm. **(e)** CUMS suppressed mice locomotors in the central arena of the open field. Unpaired two-sample *t*-test, $t(12)=9.912$, $P<0.0001$. **(f)** CUMS-treated animals had less preference for the open arm. $t(12)=3.723$, $P=0.0029$. **(g)** CUMS induced avoidance toward the light box. $t(12)=4.989$, $P=0.0003$. **(h)** CUMS mice presented significantly higher serum LCN2 levels compared to naïve ones. $t(12)=27.69$, $P<0.0001$. **(i)** LCN2 concentration in cerebrospinal fluid (CSF) was increased in CUMS mice. $t(12)=67.38$, $P<0.0001$. **(j)** Time schedule of CRS or CUMS paradigms for female age-matched mice. **(k)** No significant change of total distance in the open field. One-way ANOVA, $F(2,18)=0.8614$, $P=0.4697$. **(l)** CRS or CUMS suppressed mice locomotors in the central arena of the open field. $F(2,18)=21.57$, $P<0.0001$. **(m)** No change of locomotor activity in the elevated plus-maze. $F(2,18)=0.2279$, $P=0.9564$. **(n)** CRS or CUMS Mice displayed avoidance toward the open arm during the elevated plus-maze task. $F(2,18)=17.57$, $P<0.0001$. **(o)** Stress decreased preference of the mouse in the light box. $F(2,18)=7.845$, $P=0.0035$. **(p)** CRS or CUMS mice had significantly higher serum LCN2 levels compared to naïve ones. $F(2,18)=54.13$, $P<0.0001$. **(q)** Central LCN2 concentration was increased in stressed mice. $F(2,18)=51.57$, $P<0.0001$. $N=7$ mice in each group. All data were presented as mean±sem. Source data are provided as a Source Data file.

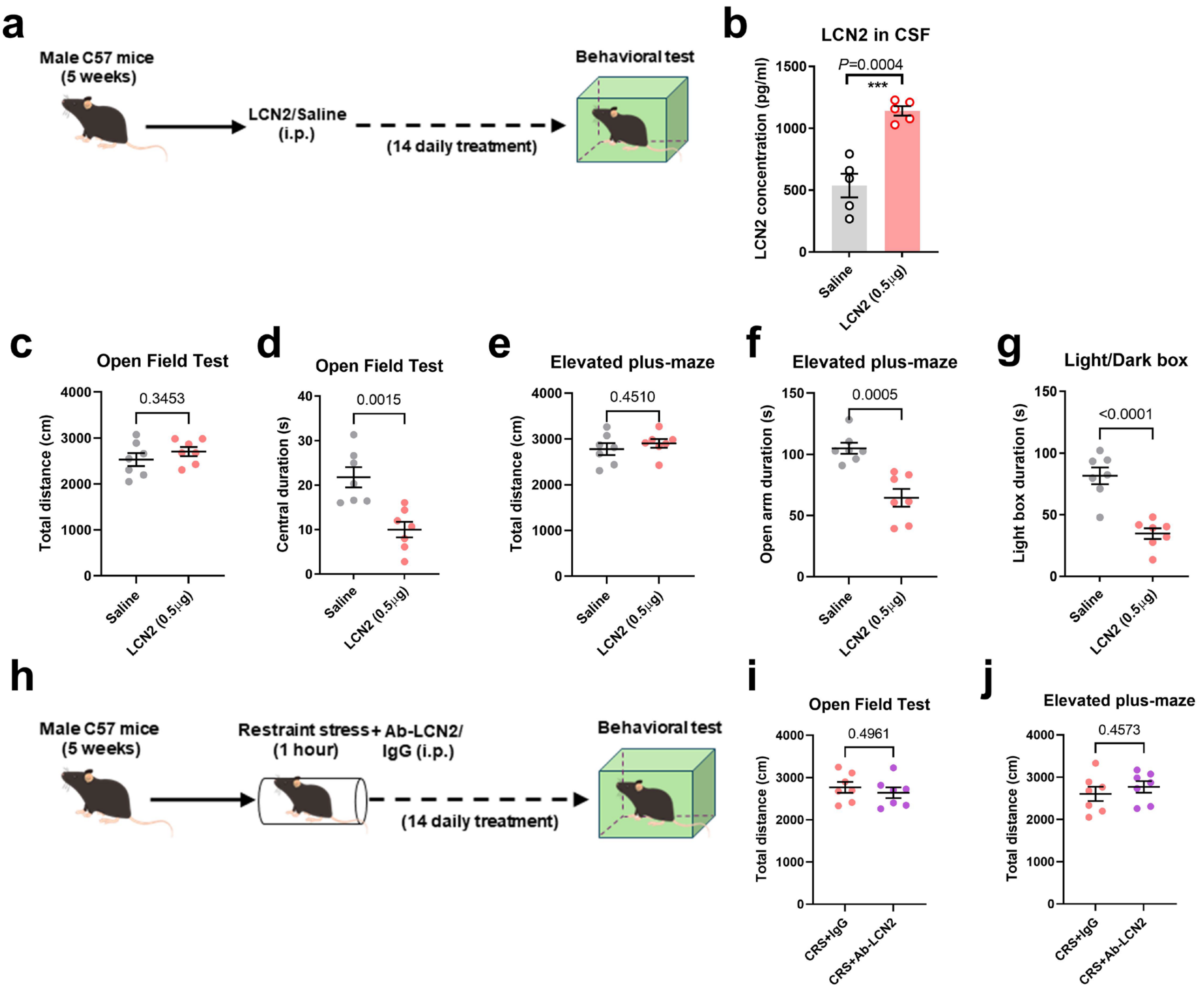

Figure S2. Peripheral LCN2 induces anxiety-like behaviors. (a) Time schedules of peripheral LCN2 activation assay, in which mice received daily LCN2 injection (0.5 μg, via intraperitoneal injection) for 14 days. **(b)** Peripheral LCN2 infusion elevated central concentrations in CSF. Two-sampled unpaired *t*-test, $t(8)=5.862$, $P=0.0004$. $N=5$ mice in each group. **(c)** Unchanged total distance in the open field. $t(12)=0.9824$, $P=0.3453$. **(d)** Peripheral LCN2 activation suppressed mice locomotors in the central arena of the open field. $t(12)=4.088$, $P=0.0015$. **(e)** Unaffected total distance in the elevated plus-maze. $t(12)=0.7791$, $P=0.4510$. **(f)** LCN2-treated animals had less preference for the open arm. $t(12)=4.706$, $P=0.0005$. **(g)** LCN2 administration induced avoidance

toward the light box. $t(12)=5.776$, $P<0.0001$. **(h)** Time schedules of peripheral LCN2 blockade assay, during which CRS mice received daily injection of polyclonal antibody against LCN2 for 14 days. **(i)** LCN2 antibody treatment did not change the total distance in the open field. $t(12)=0.7019$, $P=0.4961$. **(j)** LCN2 antibody had no effect on general motor behaviors in the elevated plus-maze. $t(12)=0.7680$, $P=0.4573$. $N=7$ mice in each group. All data were presented as mean±sem. Source data are provided as a Source Data file.

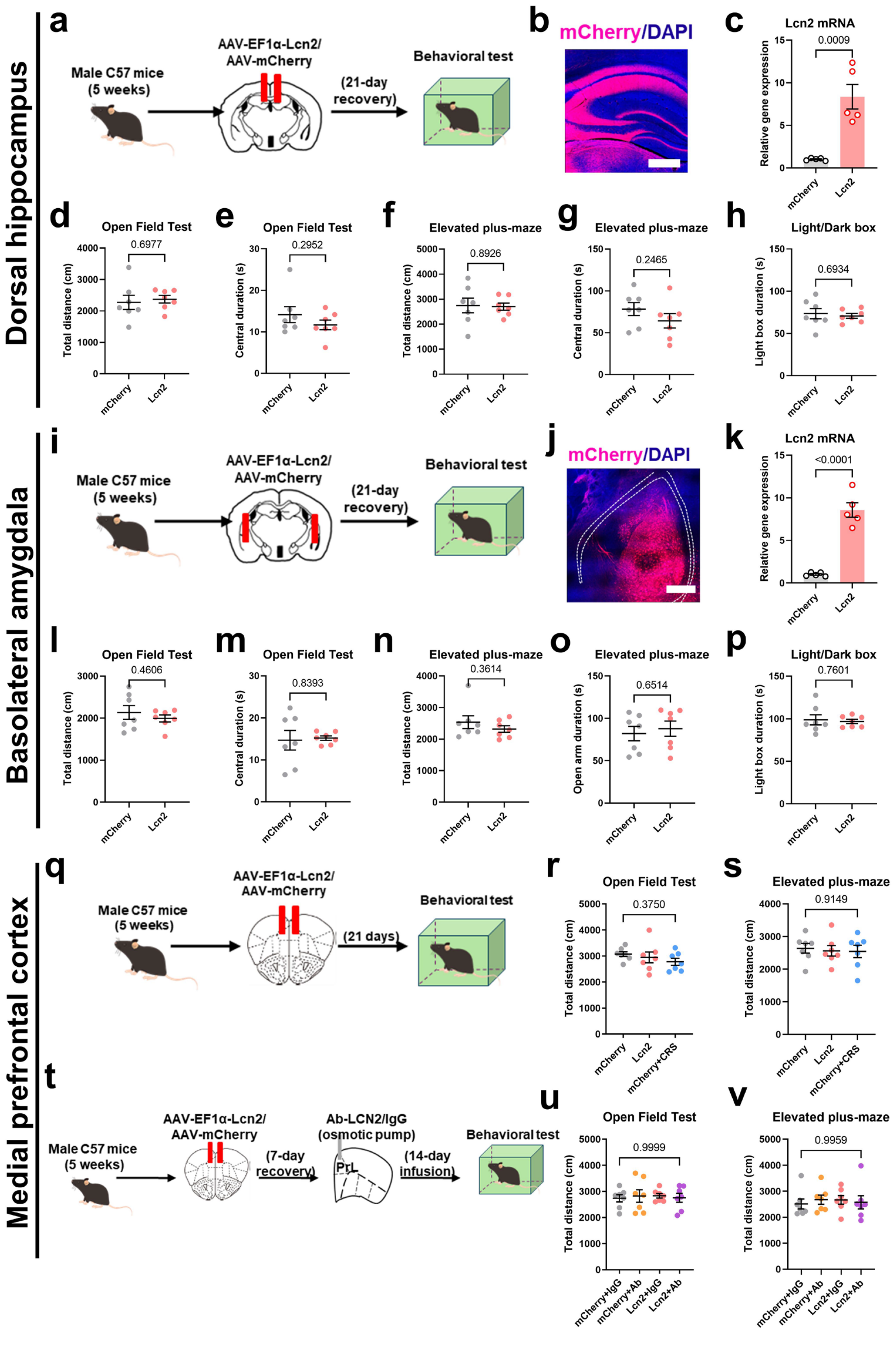

Figure S3. LCN2 did not affect anxiety-like behaviors in hippocampus or amygdala. (a)

Schematic diagram for the experimental design. **(b)** Fluorescent image showing the virus injection site of dorsal hippocampus. Scale bar, 200 μm. **(c)** Remarkably elevated *Lcn2* gene expression after viral injection. Two-sample *t*-test, $t(8)=5.098$, $P=0.0009$. $N=5$ mice in each group. **(d)** Unchanged total distance in the open field. $t(12)=0.3979$, $P=0.6977$. **(e)** Unaffected central duration after hippocampal LCN2 over-expression. $t(12)=1.095$, $P=0.2952$. **(f)** No change of total distance in the elevated plus-maze. $t(12)=0.1379$, $P=0.8926$. **(g)** LCN2 over-expression did not change the open arm duration. $t(12)=1.218$, $P=0.2465$. **(h)** No effect on the light box duration. $t(12)=0.4038$, $P=0.6934$. **(i)** Schematic diagram for the experimental design. **(j)** Fluorescent image showing the virus injection site of basolateral amygdala (BLA). Scale bar, 200 μm. **(k)** Remarkably elevated *Lcn2* gene expression after viral injection. Two-sample *t*-test, $t(8)=8.759$, $P<0.0001$. $N=5$ mice in each group. **(l)** Unchanged total distance in the open field. $t(12)=0.7632$, $P=0.4606$. **(m)** Unaffected central duration after amygdala LCN2 over-expression. $t(12)=0.2073$, $P=0.8393$. **(n)** No change of total distance in the elevated plus-maze. $t(12)=0.9488$, $P=0.3614$. **(o)** LCN2 over-expression did not change the open arm duration. $t(12)=0.4633$, $P=0.6514$. **(p)** No effect on the light box duration. $t(12)=0.3124$, $P=0.7601$. **(q)** Schematic diagrams of LCN2 overexpression in mPFC. **(r)** Lcn2 over-expression did not affect the total distance moved in the open field. One-way ANOVA, $F(2,18)=0.9495$, $P=0.4055$. **(s)** No significant change of total distance in the elevated plus-maze. $F(2,18)=0.09243$, $P=0.9121$. **(t)** Experimental outlines for LCN2 blocking assay. **(u)** No change of general locomotor activity in the open field. $F(3,24)=0.08274$, $P=0.9999$. **(v)** Undisturbed total distance on the elevated plus-maze among all groups. $F(3,24)=0.1546$, $P=0.9959$. $N=7$ mice in each group. All data were presented as mean±sem. Source data are provided as a Source Data file.

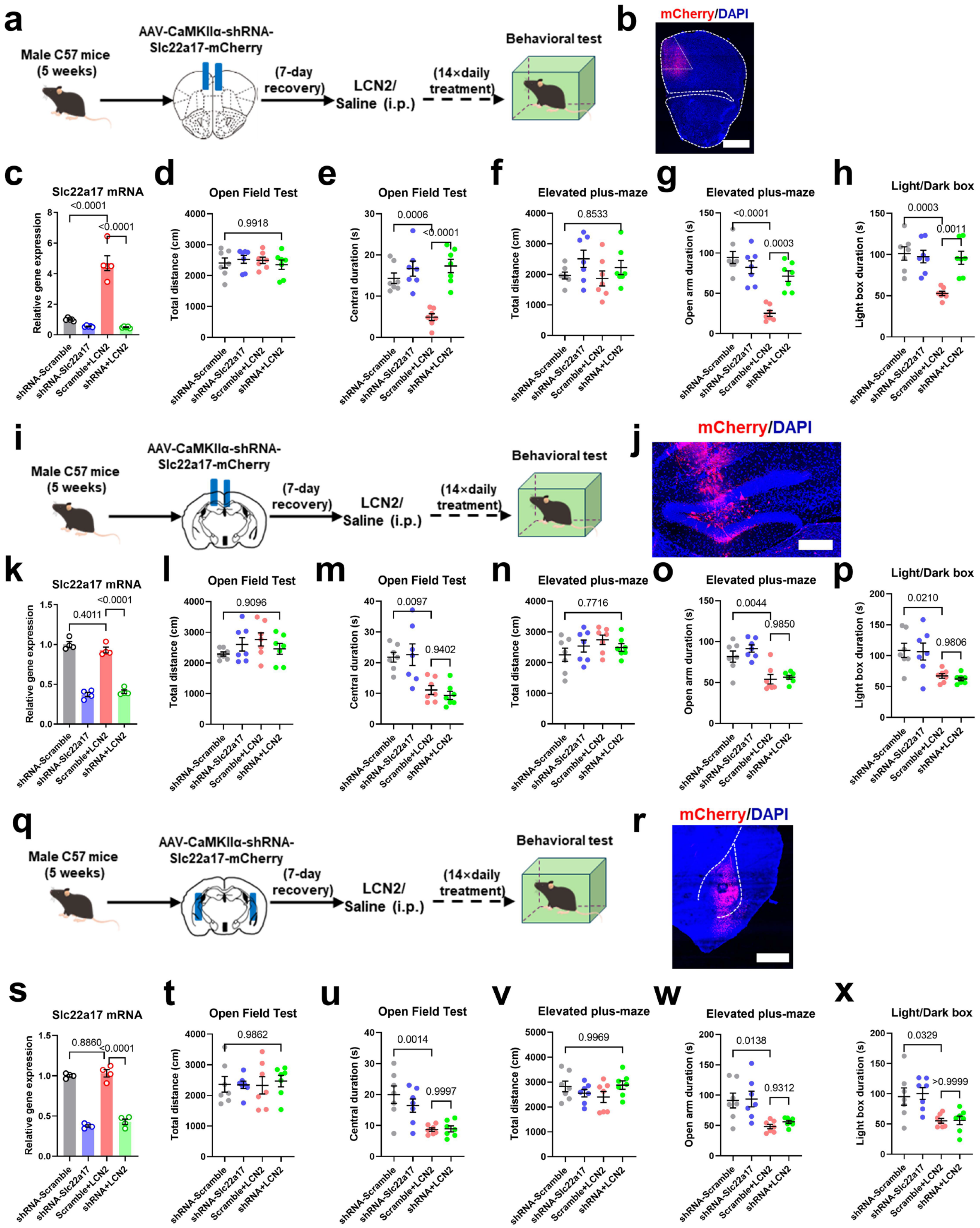

Figure S4. Circulating LCN2 induced anxiety-like behaviors via receptor binding in mPFC.

(a) Schematic illustration of experimental design of *Slc22a17* gene knockdown in mPFC. **(b)** Fluorescent image for the viral infection site. Scale bar, 500 μm. **(c)** Application of shRNA repressed local expression of *Slc22a17* gene expression. One-way ANOVA, $F(3,16)=68.16$, $P<0.0001$. $N=5$ mice in each group. **(d)** No change of general motor behaviors. $F(3,24)=0.3307$, $P=0.8302$. **(e)** Knockdown of *Slc22a17* gene in mPFC blocked the effect of peripheral LCN2 in suppressing central durations. $F(3,24)=15.93$, $P<0.0001$. **(f)** Unaffected total locomotor in the elevated plus-maze. $F(3,24)=1.604$, $P=0.2146$. **(g)** The suppression of open arm preference by LCN2 was abolished under *Slc22a17* gene knockdown in mPFC. $F(3,24)=20.64$, $P<0.0001$. **(h)** SLC22A17 deficiency also recovered normal preference toward the light box. $F(3,24)=10.77$, $P=0.0001$. $N=7$ mice in each group in **(d-h)**. **(i)** Schematic illustration of experimental design of *Slc22a17* gene knockdown in dorsal hippocampus. **(j)** Fluorescent image for the viral infection site. Scale bar, 500 μm. **(k)** Application of shRNA repressed local expression of *Slc22a17* gene expression. One-way ANOVA, $F(3,16)=118.0$, $P<0.0001$. $N=5$ mice in each group. **(l)** No change of general motor behaviors. $F(3,24)=1.276$, $P=0.3051$. **(m)** Knockdown of *Slc22a17* gene in hippocampus did not prevent the effect of peripheral LCN2 in suppressing central durations. $F(3,24)=10.28$, $P=0.0002$. **(n)** Unaffected total locomotor in the elevated plus-maze. $F(3,24)=1.311$, $P=0.2938$. **(o)** The suppression of open arm preference by LCN2 was intact under *Slc22a17* gene knockdown in hippocampus. $F(3,24)=13.04$, $P<0.0001$. **(p)** SLC22A17 deficiency did not rescue the avoidance toward the light box. $F(3,24)=7.173$, $P=0.0013$. $N=7$ mice in each group in **(l-p)**. **(q)** Schematic illustration of experimental design of *Slc22a17* gene knockdown in basolateral amygdala (BLA). **(r)** Fluorescent image for the viral infection site. Scale bar, 500 μm. **(s)** Application of shRNA repressed local expression of *Slc22a17* gene expression. One-way ANOVA, $F(3,16)=138.8$, $P<0.0001$. $N=5$

mice in each group. **(t)** No change of general motor behaviors. $F(3,24)=0.08237$, $P=0.9690$. **(u)** Knockdown of *Slc22a17* gene in BLA did not prevent the effect of peripheral LCN2 in suppressing central durations. $F(3,24)=9.108$, $P=0.0003$. **(v)** Unaffected total locomotor in the elevated plus-maze. $F(3,24)=1.403$, $P=0.2662$. **(w)** The suppression of open arm preference by LCN2 was intact under *Slc22a17* gene knockdown in BLA. $F(3,24)=6.625$, $P=0.0020$. **(x)** SLC22A17 deficiency did not rescue the avoidance toward the light box. $F(3,24)=6.491$, $P=0.0023$. $N=7$ mice in each group in **(t-x)**. Exact P values were indicated using Tukey's post-hoc comparison. All data were presented as mean±sem. Source data are provided as a Source Data file.

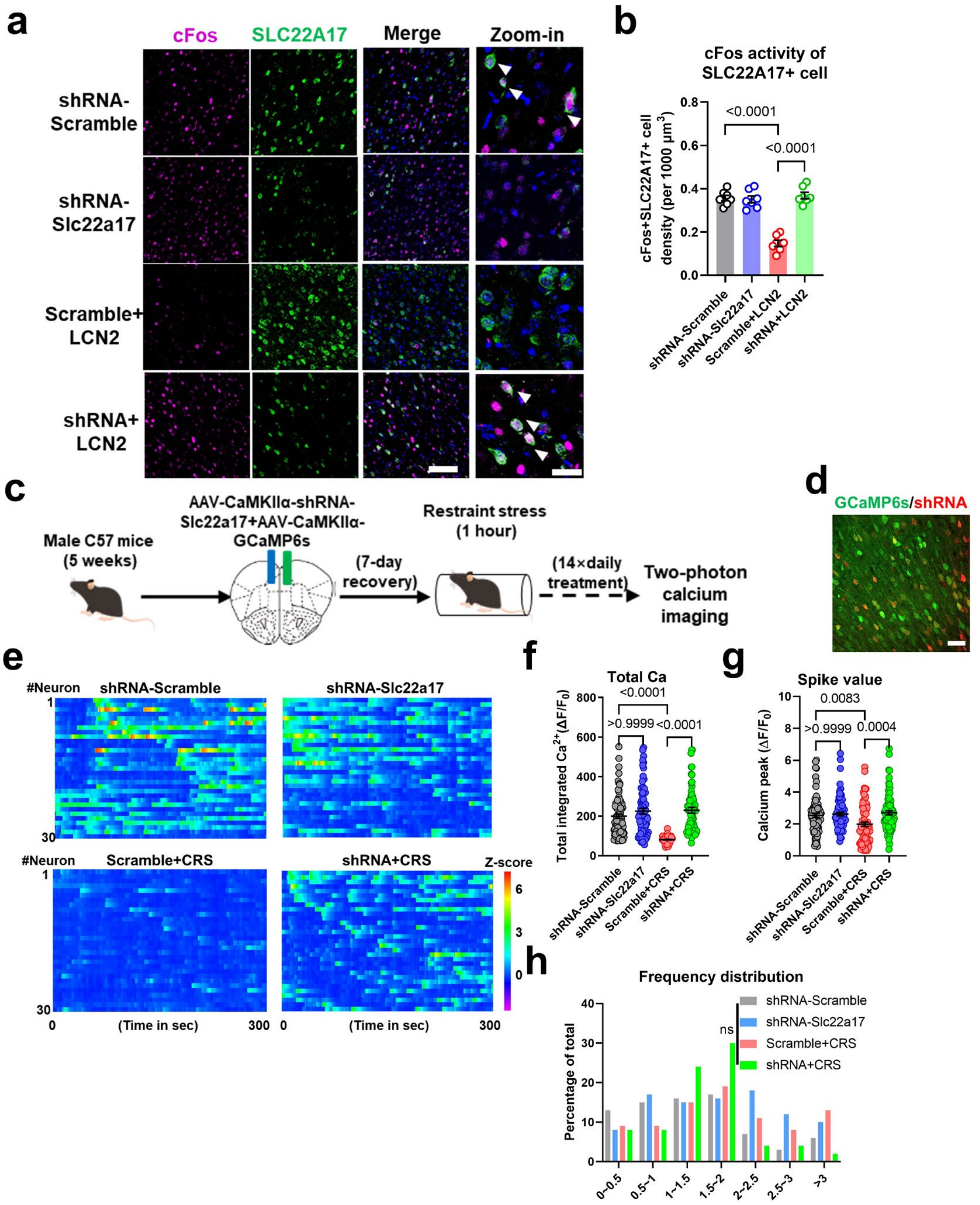

Figure S5. LCN2-SLC22A17 axis mediates cortical neuronal activity. (a) Immunofluorescent staining of cFos in SLC22A17+ neurons in PrL. Scale bar, 150 μm and 50 μm for zoom-in images. **(b)** Quantification of cFos in SCL22A17+ cells. LCN2 effectively suppressed neuronal activity of those cells expressing its receptor. One-way ANOVA, $F(3,24)=54.44$, $P<0.0001$. $N=7$ mice each group. **(c)** Outlines of LCN2 receptor blockade assay, in which mice received shRNA targeting *Slc22a17* gene into bilateral mPFC, followed by 14-day CRS. **(d)** Co-expression of GCaMP6s and

shRNAS. Scale bar, 100 μm. **(e)** Heatmaps of mPFC PNs during the *in vivo* imaging session, using the same data processing approach as in **Fig. 3c**. **(f)** CRS-induced hypoactivity of mPFC neurons was reversed by down-regulation of SLC22A17. Nonparametric Kruskal-Wallis test statistic=147.9, P<0.0001. **(g)** With no change of basal level calcium peak values, *Slc22a17* gene knockdown in mPFC prevented the suppression of PNs activity. Kruskal-Wallis test statistic=20.54, P=0.0001. **(h)** Frequency distribution of calcium transient frequency. n=80 neurons (from 4 animals) in each group in **(m-o)**. Exact P values were indicated using Tukey's post-hoc comparison in **(b)**, and Dunn's multiple comparison test in **(f-g)**. All data were presented as mean±sem. Source data are provided as a Source Data file.

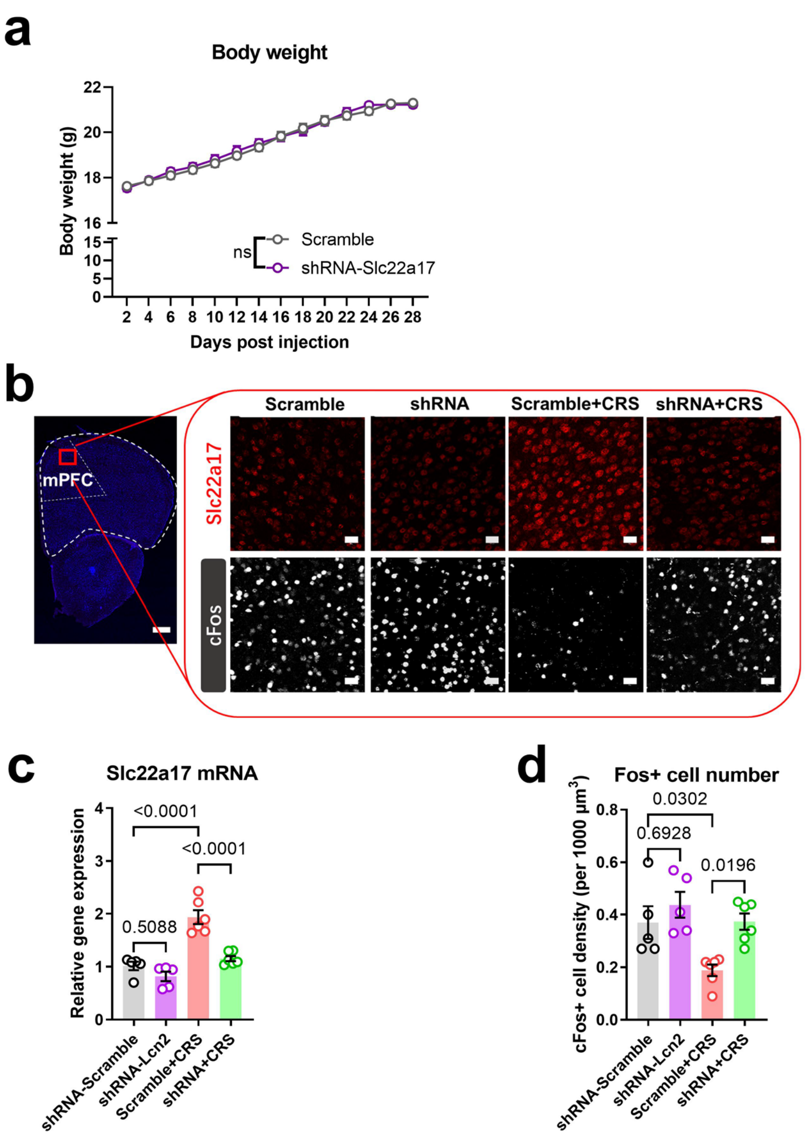

Figure S6. Hepatic LCN2 deficiency affected brain function. (a) No change of body weight after injecting liver-target AAV vector for 28 days. ns, no significant difference. Two-way ANOVA with respect to day × group interaction effect, $F(13, 156)=0.8622$, $P=0.5940$. $N=7$ mice in each group. **(b)** Immunofluorescent images for SLC22A17 and cFos. Scale bar, 150 μm in the left and 50 μm in the right panels. **(c)** *Slc22a17* gene expression was downregulated by shRNA. One-way ANOVA, $F(3,18)=28.10$, $P<0.0001$. **(d)** The decreased mPFC neuronal activity under CRS was recovered by shRNA injection. $F(3,18)=6.900$, $P=0.0027$. $N=5$ mice in each group in **(c-d)**. Exact P values were

indicated using Tukey's post-hoc comparison in **(c-d)**. All data were presented as mean ± sem. Source data are provided as a Source Data file.

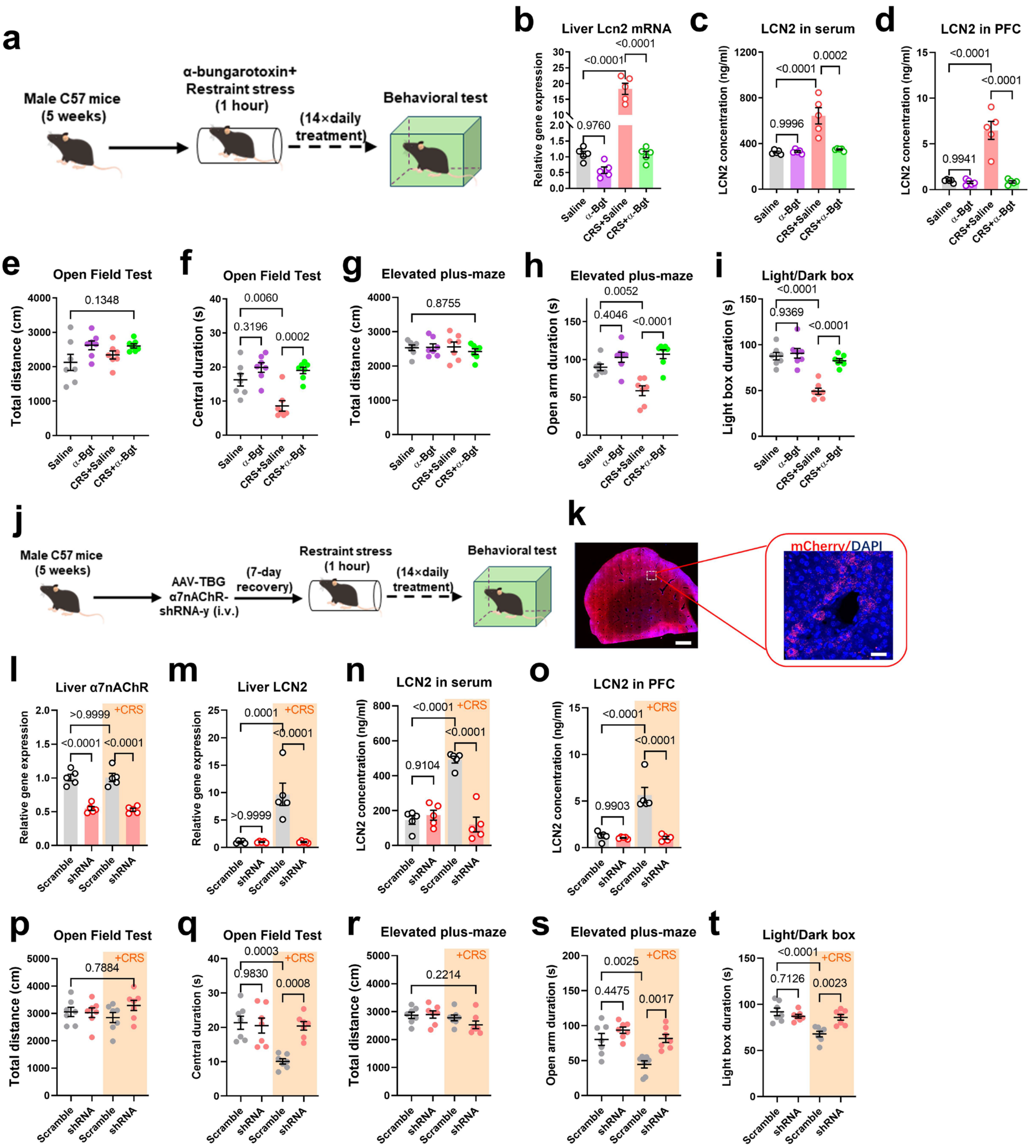

Figure S7. Nicotinic acetylcholine receptor in vagal pathway mediates hepatic LCN2 production and stress-induced anxiety-like behaviors. (a) Experimental design of pharmaceutical inhibition. The antagonist of α7 subunit of nicotinic acetylcholine receptor, α-bungarotoxin, was injected in CRS mice. **(b)** Blockade of acetylcholine receptor reversed hepatic

LCN2 expression in CRS mice. One-way ANOVA, $F(3,16)=96.77$, $P<0.0001$. **(c)** Serum LCN2 level was unchanged in naïve mice even with α-bungarotoxin, whilst CRS-induced LCN2 surge was repressed. $F(3,16)=18.22$, $P<0.0001$. **(d)** LCN2 in PFC was also elevated under CRS and was decreased after nicotinic acetylcholine receptor blockade. $F(3,16)=31.39$, $P<0.0001$. $N=5$ mice per group in **(b-d)**. **(e)** No effect of α-Bgt on locomotor distance in naïve or CRS mice. $F(3,24)=2.502$, $P=0.0838$. **(f)** Blockade of acetylcholine receptor did not change central zone duration in naïve animals, but relieved CRS-induced avoidance phenotype. $F(3,24)=12.21$, $P<0.0001$. **(g)** No change of total distance in the elevated plus-maze. $F(3,24)=0.3700$, $P=0.7754$. **(h)** α-Bgt also enhanced open arm preference in CRS mice. $F(3,24)=13.92$, $P<0.0001$. **(i)** The avoidance toward the light box under CRS was recovered under acetylcholine receptor blocking. $F(3,24)=24.18$, $P<0.0001$. $N=7$ mice in each group in **(e-i)**. **(j)** Experimental design of genetic blockade of α7nAChR using an AAV-mediated shRNA vector. **(k)** Hepatic viral infection site. Scale bar, 25 μm. **(l)** The knockdown of α7nAChR gene expression. $F(3,16)=39.61$, $P<0.0001$. **(m)** Hepatic deficiency of acetylcholine receptor effectively decreased hepatic LCN2 expression in CRS but not naïve mice. $F(3,16)=17.96$, $P<0.0001$. **(n)** Serum LCN2 level was unchanged in naïve with α7nAChR gene knockdown, whilst CRS-induced LCN2 surge was repressed. $F(3,16)=33.90$, $P<0.0001$. **(o)** LCN2 in PFC was also elevated under CRS and was decreased after nicotinic acetylcholine receptor gene knockdown. $F(3,16)=27.10$, $P<0.0001$. $N=5$ mice per group in **(l-o)**. **(p)** No effect of α7nAChR gene knockdown on locomotor distance in naïve or CRS mice. $F(3,24)=1.026$, $P=0.3988$. **(q)** Deficiency of acetylcholine receptor did not change central zone duration in naïve animals, but relieved CRS-induced avoidance phenotype. $F(3,24)=10.80$, $P=0.0001$. **(r)** No change of total distance in the elevated plus-maze. $F(3,24)=1.896$, $P=0.1572$. **(s)** α7nAChR gene knockdown also enhanced open

arm preference in CRS mice. $F(3,24)=11.53$, $P<0.0001$. **(t)** The avoidance toward the light box under CRS was recovered under acetylcholine receptor deficiency. $F(3,24)=11.30$, $P<0.0001$. $N=7$ mice in each group in **(p-t)**. Exact P values were indicated using Tukey's post-hoc comparison. All data were presented as mean±sem. Source data are provided as a Source Data file.

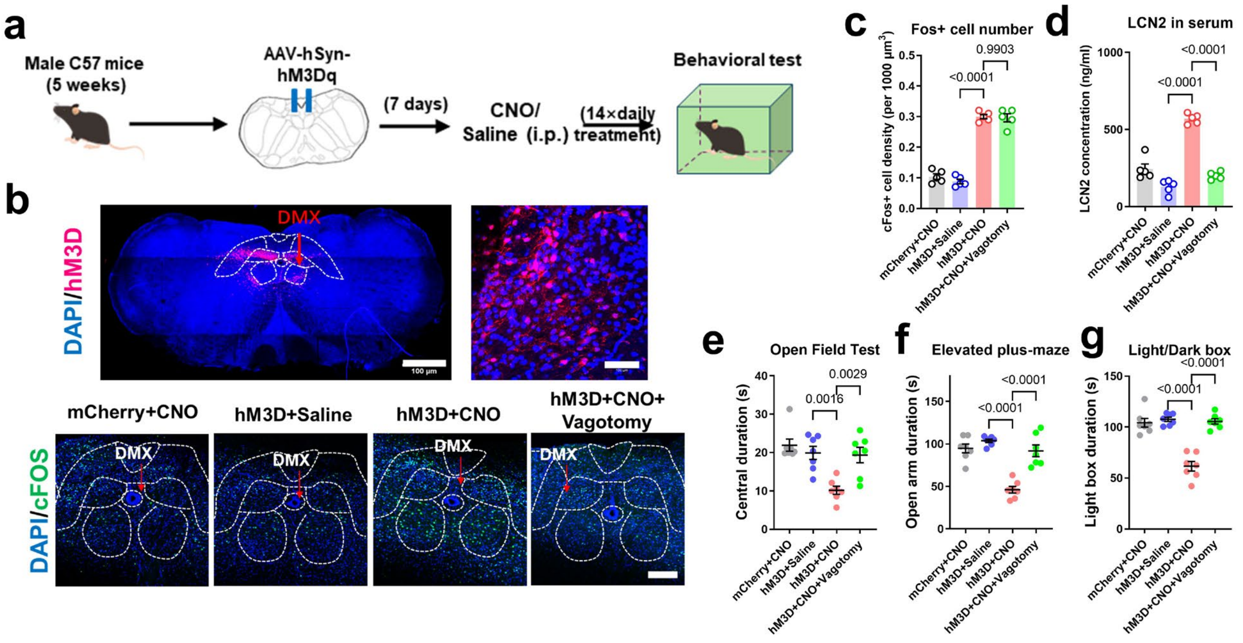

Figure S8. DMX stimulates circulating LCN2 via vagal pathway. (a) Schematic diagrams for chemogenetic activation assay, in which excitatory receptor hM3Dq was transfected into DMX, followed by CNO infusion. **(b)** Upper, viral transfection site. Lower, cFos staining under CNO infusion. Scale bars, 100 μm. **(c)** Quantification of Fos activity showed neuronal activation upon CNO infusion. One-way ANOVA, $F(3,16)=153.3$, $P<0.0001$. **(d)** Chemogenetic activation of DMX elevated serum LCN2 level, which was abolished by vagotomy. $F(3,16)=86.96$, $P<0.0001$. $N=5$ mice in each group in **(c-d)**. **(e)** Neuronal activation in DMX resulted in lower central zone duration in naïve mice, and such effects can be blocked by vagotomy. $F(3,24)=10.30$, $P=0.0002$. **(f)** Decreased preference for the open arm after DMX activation, plus the restoration under vagotomy. $F(3,24)=28.35$, $P<0.0001$. **(g)** Lower duration in the light box in DMX-activated animals in a vagal-dependent manner. $F(3,24)=37.94$, $P<0.0001$. $N=7$ mice in each group in **(e-g)**. Exact P values were indicated using Tukey's post-hoc comparison. All data were presented as mean±sem. Source data are provided as a Source Data file.

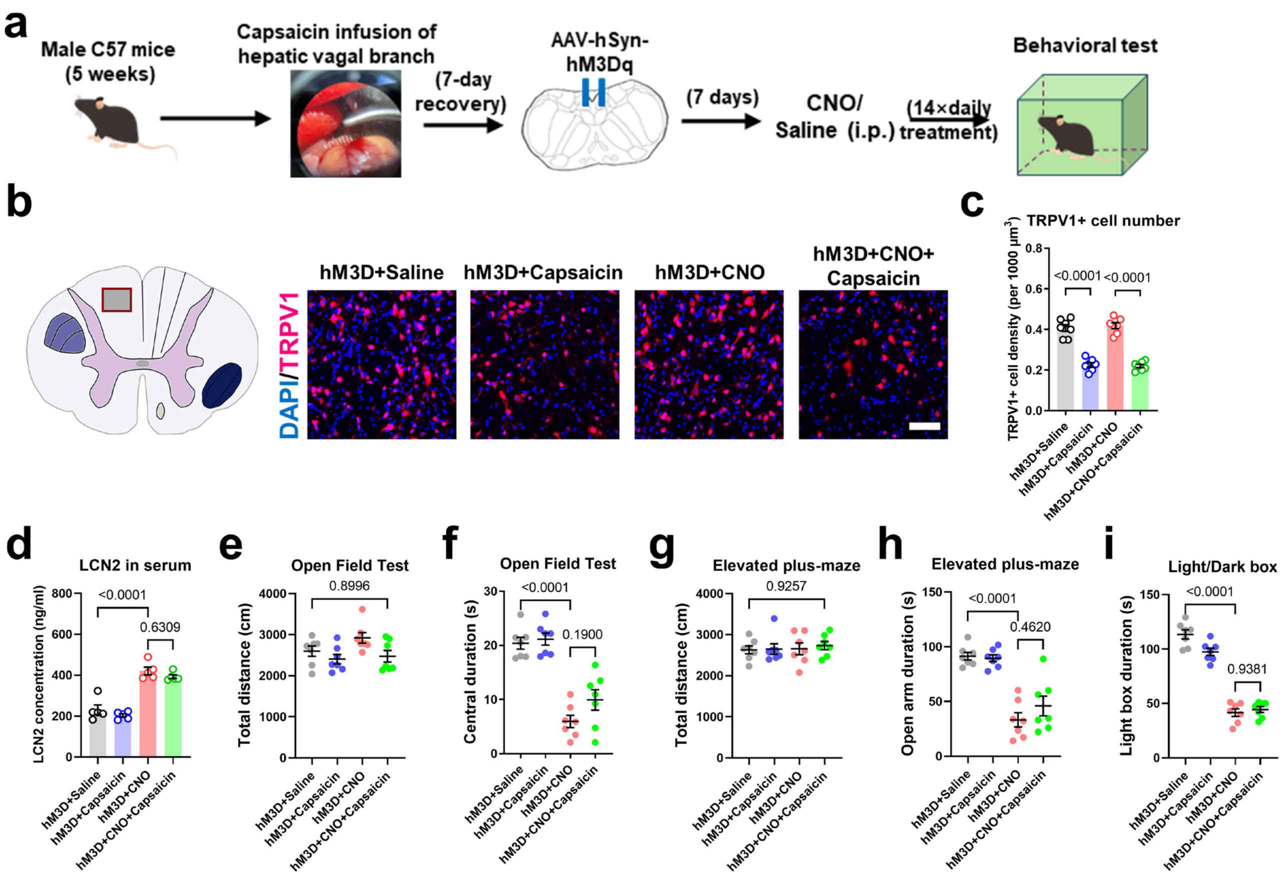

Figure S9. Vagal afferent pathway is unrelated with LCN2-medaited anxiety behaviors. (a) Schematic diagram of local delivery of capsaicin onto hepatic vagal branch for deafferentation, followed by DMX-activation to induce hepatic LCN2. **(b)** Immunofluorescent staining of TRPV1+ neurons in dorsal spinal cords (grey box). Scale bar, 100 μm. **(c)** Decreased density of TRPV1+ cells by capsaicin treatment. One-way ANOVA, $F(3,24)=70.85$, $P<0.0001$. $N=7$ mice per group. **(d)** Capsaicin did not alter the blood LCN2 surge caused by DMX-activation. $F(3,16)=45.21$, $P<0.0001$. $N=5$ mice each group. **(e)** No change of total distance in the open field. $F(3,24)=2.263$, $P=0.0789$. **(f)** Capsaicin did not rescue the decreased central zone duration in DMX-activation group. $F(3,24)=31.50$, $P<0.0001$. **(g)** Unaffected distance in the elevated plus-maze. $F(3,24)=0.1504$, $P=0.9284$. **(h)** The avoidance toward the open arm was not improved by blocking vagal afferent pathway. $F(3,24)=24.39$, $P<0.0001$. **(i)** No effect on DMX-activation induced light box avoidance

by capsaicin treatment. $F(3,24)=109.6$, $P<0.0001$. $N=7$ mice in each group in **(e-i)**. Exact P values were indicated using Tukey's post-hoc comparison in **(c-i)**. All data were presented as mean±sem. Source data are provided as a Source Data file.

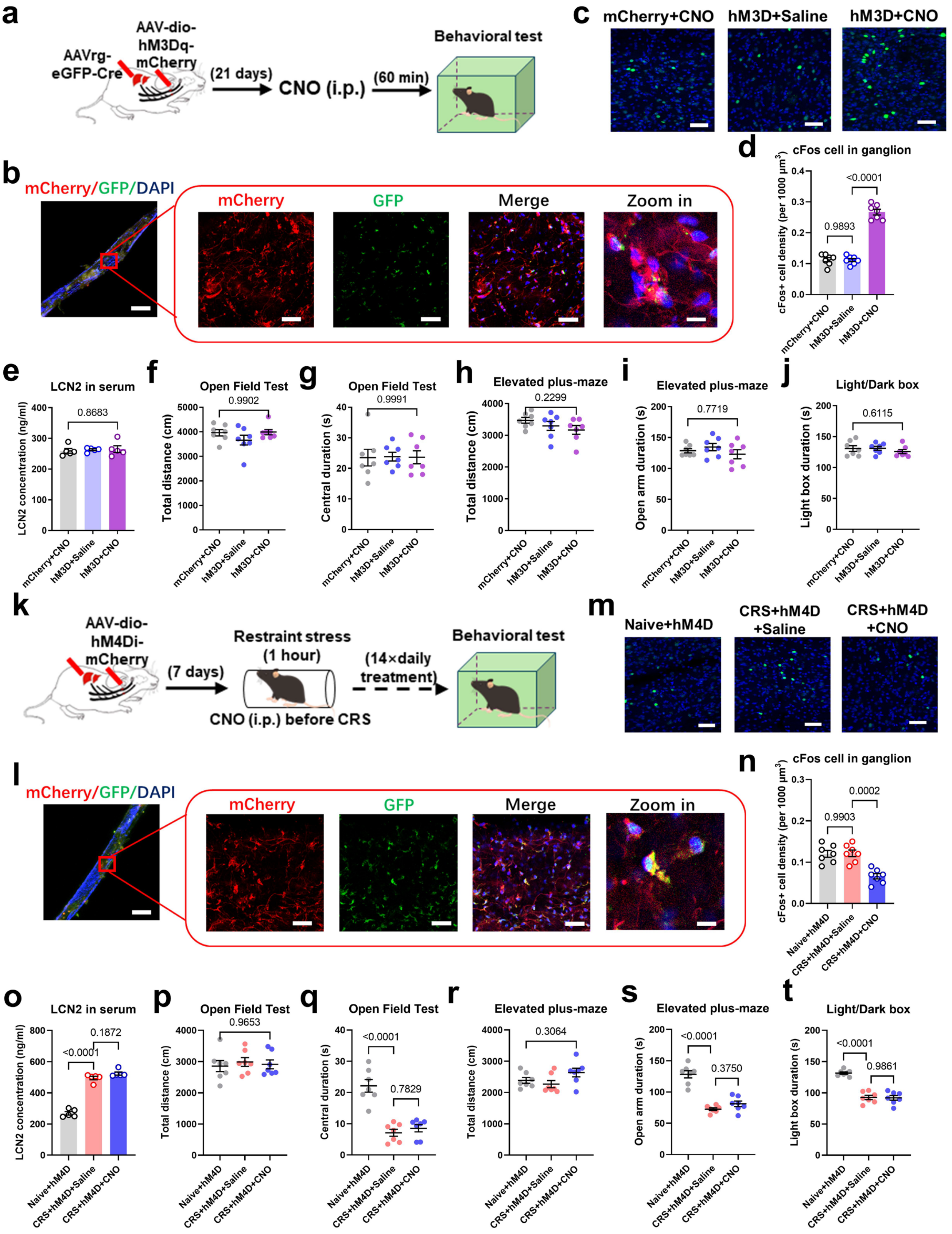

Figure S10. Nodose ganglia did not mediate CRS-induced LCN2 release or anxiety-like

behaviors. (a) Timelines for specific labelling and chemogenetic activation of liver-mediated vagal afferent neurons in nodose ganglia. **(b)** Viral infection stie within the nodose ganglia. Scale bar, 500 μm (left), 100 μm (right) and 10 μm (right, zoom in). **(c)** Fluorescent images of hM3Dq expression in ganglion. Scale bar, 150 μm. **(d)** Density of cFos+ cells. One-way ANOVA, $F(2,18)=153.6$, $P<0.0001$. $N=7$ mice per group. **(e)** Chemogenetic activation did not affect serum LCN2 level. $F(2,12)=0.1310$, $P=0.8784$. $N=5$ mice per group. **(f)** Nodose ganglia activation did not affect the general motor in the open field. $F(2,18)=1.486$, $P=0.2526$. **(g)** Nodose ganglia activation did not change the central duration. $F(2,18)=0.06881$, $P=0.9931$. **(h)** Nodose ganglia activation did not change the total distance on the elevated plus-maze. $F(2,18)=1.469$, $P=0.2565$. **(i)** Nodose ganglia activation did not alter the time spent in the open arm. $F(2,18)=1.010$, $P=0.3839$. **(j)** Nodose ganglia activation did not change the preference toward the light box. $F(2,18)=0.6576$, $P=0.5031$. $N=7$ mice per group in **(f-j)**. **(k)** Timelines for chemogenetic inhibition assays. **(l)** Viral infection stie within nodose ganglia. Scale bar, 500 μm (left), 100 μm (right) and 10 μm (right, zoom in). **(m)** Fluorescent images of hM4Di expression in ganglion. Scale bar, 150 μm. **(n)** Density of cFos+ cells. $F(2,18)=17.52$, $P<0.0001$. $N=7$ mice per group. **(o)** Chemogenetic inhibition failed to attenuate serum LCN2 surge in CRS mice. One-way ANOVA, $F(2,12)=165.5$, $P<0.0001$. $N=5$ mice per group. **(p)** Nodose ganglia inhibition did not affect the general motor in the open field. $F(2,18)=0.1827$, $P=0.8346$. **(q)** Nodose ganglia inhibition cannot prevent the avoidance toward the central region. $F(2,18)=30.75$, $P<0.0001$. **(r)** Nodose ganglia inhibition did not change the total distance on the elevated plus-maze. $F(2,18)=2.610$, $P=0.1011$. **(s)** Nodose ganglia activation did not recover normal time spent in the open arm in CRS mice. $F(2,18)=46.13$, $P<0.0001$. **(t)** Nodose ganglia inhibition did not reverse the avoidance toward the light box.

$F(2,18)=54.34$, $P<0.0001$. $N=7$ mice per group in **(p-t)**. Exact *P* values were indicated using Tukey's post-hoc comparison. All data were presented as mean±sem. Source data are provided as a Source Data file.

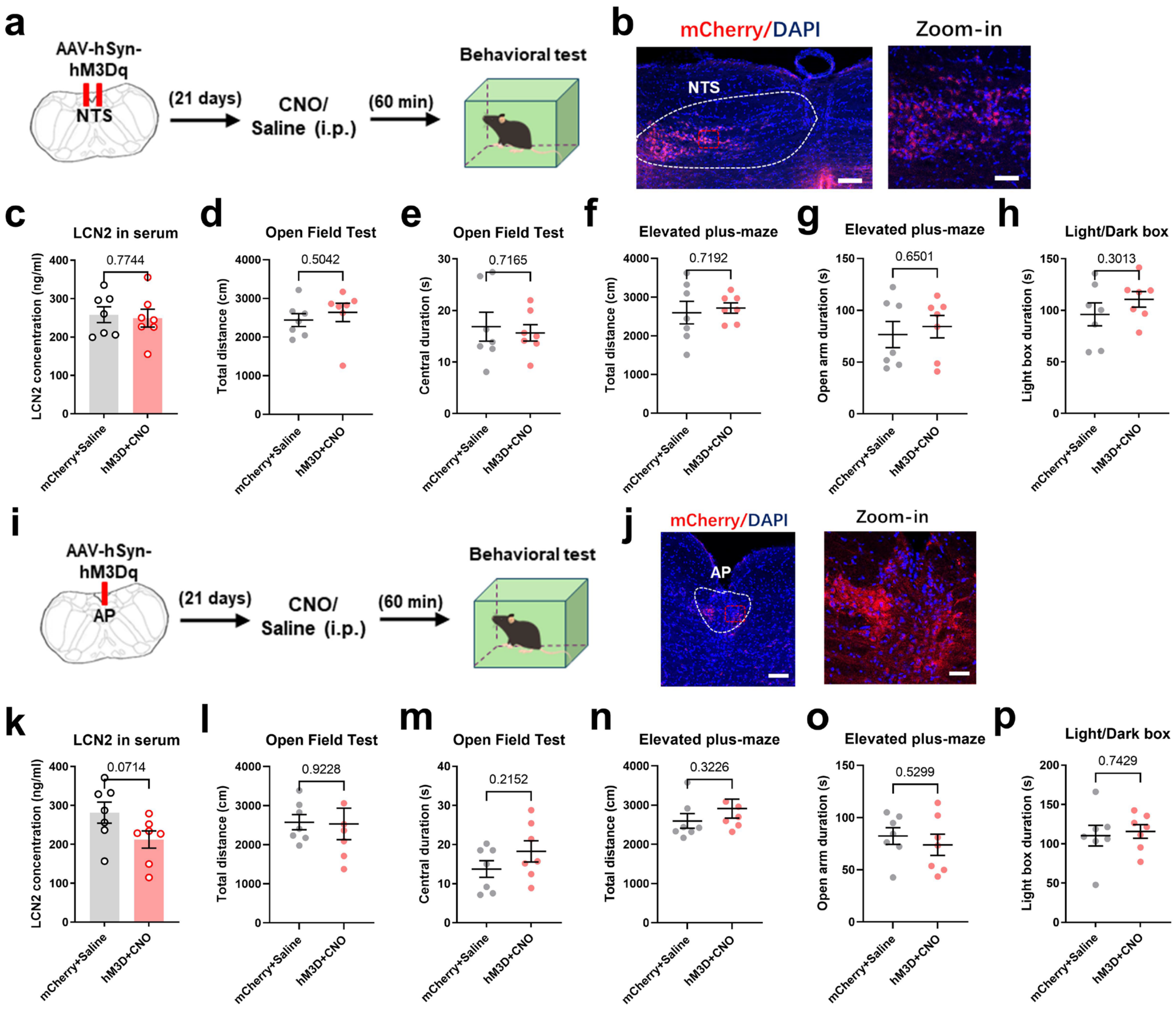

Figure S11. Other vagal afferent projecting brain regions did not affect LCN2 release or anxiety-like behaviors. (a) Timelines for chemogenetic activation of NTS. **(b)** Viral infection stie within the NTS. Scale bar, 200 μm (left) and 50 μm (right). **(c)** Chemogenetic activation did not affect serum LCN2 level. Two-sampled unpaired *t*-test, $t(12)=0.2932$, $P=0.7744$. **(d)** NTS activation did not affect the general motor in the open field. $t(12)=0.6886$, $P=0.5042$. **(e)** NTS activation did not change the central duration. $t(12)=0.3719$, $P=0.7165$. **(f)** NTS activation did not change the total distance on the elevated plus-maze. $t(12)=0.3681$, $P=0.7192$. **(g)** NTS activation did not alter the time spent in the open arm. $t(12)=0.4652$, $P=0.6501$. **(h)** NTS activation did not change the

preference toward the light box. $t(12)=1.080$, $P=0.3013$. **(i)** Timelines for chemogenetic activation of AP. **(j)** Viral infection stie within the AP. Scale bar, 200 μm (left) and 50 μm (right). **(k)** Chemogenetic activation did not affect serum LCN2 level. $t(12)=1.977$, $P=0.0714$. **(l)** AP activation did not affect the general motor in the open field. $t(12)=0.09894$, $P=0.9288$. **(m)** AP activation did not change the central duration. $t(12)=1.309$, $P=0.2152$. **(n)** AP activation did not change the total distance on the elevated plus-maze. $t(12)=1.032$, $P=0.3226$. **(o)** AP activation did not alter the time spent in the open arm. $t(12)=0.6469$, $P=0.5299$. **(p)** AP activation did not change the preference toward the light box. $t(12)=0.3357$, $P=0.7429$. $N=7$ mice per group. All data were presented as mean ±sem. Source data are provided as a Source Data file.

Table S1. Demographic information of MDD and healthy control people cohort.

Healthy control group			MDD patient group		
**Doc. No.**	**Gender**	**Age (yrs)**	**Doc. No.**	**Gender**	**Age (yrs)**
C201	Female	24	P203	Female	22
C202	Female	23	P208	Female	30
C203	Female	26	P214*	Female	31
C204	Female	23	P215*	Female	28
C206	Female	28	P228*	Female	24
C405	Female	32	P238	Female	23
C406	Female	29	P426*	Female	29
C101	Male	23	P431*	Female	33
C102	Male	29	P106	Male	32
C176	Male	30	P113*	Male	24
C196	Male	27	P128*	Male	25
C410	Male	23	P418*	Male	29
C412	Male	34	P420*	Male	33
Total Male=6 (46%), Female=7 (54%)			Total Male=5 (38%), Female=8 (62%)		

Average age =27.0 yrs in Healthy, and 27.9 yrs in MDD,
P=0.543 using Two-sided unpaired *t*-test.

Patients with * were enrolled in the secondary blood sampling after treatment.

Table S2 List of antibody and virus used in the experiments.

REAGENT or RESOURCE	SOURCE	IDENTIFIER or SEROTYPE	APPLICATION REGION/ROUTE	FIGURES
**Primary Antibody**				
GAPDH Antibody	CST	5174s		
Lcn2 Antibody	Invitrogen	PA5-79590	i.p. injection Immuno-fluorescence	Fig. 1k-p; Fig. 4d-e; Fig. 5f; Fig. S2h-j
Slc22a17 Antibody	Invitrogen	PA5-20543	Immuno-fluorescence	Fig. 2a Fig. 3h-I; Fig. 5j
Rabbit Anti-c-Fos	Cell Signaling Technology	Cat# 2250; RRID: AB_2247211	Immuno-fluorescence	Fig. 6b-d Fig. S5a-b
ChAT Antibody	Millipore	Cat# AB144-P	Immuno-fluorescence	Fig. 6b-d
**Viral vectors**				
rAAV-U6-shRNA (Slc22a17)-CMV-EGFP-SV40 pA	Wuhan BrainVTA Co., Ltd.	AAV2/8	Stereotaxic injection to PrL; Stereotaxic injection to hippocampus & BLA	Fig. 3j-I; Fig. S4; Fig. S5c-h; Fig. S6
rAAV-U6-shRNA(scramble)-CMV-EGFP-SV40 pA	Wuhan BrainVTA Co., Ltd.	AAV2/8	Stereotaxic injection to PrL; Stereotaxic injection to hippocampus & BLA	Fig. 3j-I; Fig. S4; Fig. S5c-h; Fig. S6
rAAV -EF1α-P2A-Lcn2-mCherry-WPRE-hGH	Wuhan BrainVTA Co., Ltd.	AAV2/9	Stereotaxic injection to PrL; Stereotaxic injection to hippocampus & BLA	Fig. 2, Fig. 3a-f Fig. S3
rAAV -EF1α-P2A-mCherry-WPRE-hGH	Wuhan BrainVTA Co., Ltd.	AAV2/9	Stereotaxic injection to PrL; Stereotaxic injection to hippocampus &	Fig. 2, Fig. 3a-f Fig. S3

			BLA	
RV-ENVA-ΔG-EGFP	Wuhan BrainVTA Co., Ltd.	AAV2/9	Injection to liver tissues	Fig. 6a
rAAV-TBG-mCherry-5'miR-30a-shRNA(Lcn2)-3'-miR30a-WPREs	Wuhan BrainVTA Co., Ltd.	AAV2/8	i.v. injection	Fig. 4c-p
rAAV-TBG-mCherry-5'miR-30a-shRNA(scramble)-3'-miR30a-WPREs	Wuhan BrainVTA Co., Ltd.	AAV2/8	i.v. injection	Fig. 4c-p
rAAV-CaMKIIa-GCaMP6s-WPRE-hGH pA	Wuhan BrainVTA Co., Ltd.	AAV2/9	Stereotaxic injection to PrL	Fig. 3; Fig. 4c-p; Fig. S5c-h
rAAV-CRE-WPREs	Wuhan BrainVTA Co., Ltd.	AAV2/9	Injection to liver tissues	Fig. S10
rAAV- hSyn-hM4D(Gi)-mCherry	Taitool BioScience Co, Shanghai	AAV2/9	Stereotaxic injection to DMX	Fig. 7h-n
rAAV- hSyn-hM3D(Gi)-mCherry	Taitool BioScience Co, Shanghai	AAV2/9	Stereotaxic injection to DMX; Stereotaxic injection to NTS&AP	Fig. 7a-g; Fig. S8; Fig. S9; Fig. S11
rAAV- hSyn-mCherry	Taitool BioScience Co, Shanghai	AAV2/9	Stereotaxic injection to DMX; Stereotaxic injection to NTS&AP	Fig. 7; Fig. S8; Fig. S9; Fig. S11
rAAV-WGA-CRE-P2A-mCherry-WPREs	Wuhan BrainVTA Co., Ltd.	AAV2/9	Stereotaxic injection to DMX	Fig. 6e-r
rAAV-CAG-DIO-eNpHR3.0-EYFP-WPRE-pA	Wuhan BrainVTA Co., Ltd.	AAV2/9	Stereotaxic injection to DMX	Fig. 6l-r
rAAV-CAG-DIO-hChR2(H134R)-EYFP-WPRE-pA	Wuhan BrainVTA Co., Ltd.	AAV2/9	Stereotaxic injection to DMX	Fig. 6e-k
rAAV-Ef1α-DIO-hM3D(Gq)-mCherry-WPREs	Wuhan BrainVTA Co., Ltd.	AAV2/9	Stereotaxic injection to DMX	Fig. S10a-j
rAAV-Ef1α-DIO-hM4D(Gi)-	Wuhan BrainVTA Co., Ltd.	AAV2/9	Stereotaxic injection to DMX	Fig. S10k-t

mCherry-WPREs				
rAAV-TBG-mCherry-5'miR-30a-shRNA(α7nAChR)-3'-miR30a-WPREs	Wuhan BrainVTA Co., Ltd.	AAV2/9	Injection to liver tissues	Fig. S7j-t
