# Supplementary material for: Stress increases hepatic release of lipocalin 2 which contributes to anxiety-like behavior in mice
Source: Nat Commun. 2024 Apr 8;15:3034. doi: 10.1038/s41467-024-47266-9 (PMC11001612; doi:10.1038/s41467-024-47266-9)
Supplement: Supplementary file 3 — Reporting Summary [file 41467_2024_47266_MOESM3_ESM.pdf]

nature portfolio

Corresponding author(s): Li Zhang

Last updated by author(s): Mar 8, 2024

Reporting Summary

Nature Portfolio wishes to improve the reproducibility of the work that we publish. This form provides structure for consistency and transparency in reporting. For further information on Nature Portfolio policies, see our Editorial Policies and the Editorial Policy Checklist.

Statistics

For all statistical analyses, confirm that the following items are present in the figure legend, table legend, main text, or Methods section.

n/a	Confirmed	
☐	☒	The exact sample size (*n*) for each experimental group/condition, given as a discrete number and unit of measurement
☐	☒	A statement on whether measurements were taken from distinct samples or whether the same sample was measured repeatedly
☐	☒	The statistical test(s) used AND whether they are one- or two-sided<br>*Only common tests should be described solely by name; describe more complex techniques in the Methods section.*
☒	☐	A description of all covariates tested
☐	☒	A description of any assumptions or corrections, such as tests of normality and adjustment for multiple comparisons
☐	☒	A full description of the statistical parameters including central tendency (e.g. means) or other basic estimates (e.g. regression coefficient) AND variation (e.g. standard deviation) or associated estimates of uncertainty (e.g. confidence intervals)
☐	☒	For null hypothesis testing, the test statistic (e.g. *F*, *t*, *r*) with confidence intervals, effect sizes, degrees of freedom and *P* value noted<br>*Give P values as exact values whenever suitable.*
☒	☐	For Bayesian analysis, information on the choice of priors and Markov chain Monte Carlo settings
☒	☐	For hierarchical and complex designs, identification of the appropriate level for tests and full reporting of outcomes
☒	☐	Estimates of effect sizes (e.g. Cohen's *d*, Pearson's *r*), indicating how they were calculated

Our web collection on statistics for biologists contains articles on many of the points above.

Software and code

Policy information about availability of computer code

Data collection	Zen (v8.1.0.484, Zeiss Inc.), Bio-Rad CFX Maestro (v1.0, BIO-RAD Inc.), Ethovision XT (v8.0, Noldus Inc.)
Data analysis	Excel (in Microsoft 365 package), Prism (v9.4, GraphPad), ImageJ (v1.52, NIH)

For manuscripts utilizing custom algorithms or software that are central to the research but not yet described in published literature, software must be made available to editors and reviewers. We strongly encourage code deposition in a community repository (e.g. GitHub). See the Nature Portfolio guidelines for submitting code & software for further information.

Data

Policy information about availability of data

All manuscripts must include a data availability statement. This statement should provide the following information, where applicable:

- Accession codes, unique identifiers, or web links for publicly available datasets
- A description of any restrictions on data availability
- For clinical datasets or third party data, please ensure that the statement adheres to our policy

All data generated or analyzed during this study are included in this published article (and its supplementary information files). Source data are provided with this paper.

Research involving human participants, their data, or biological material

Policy information about studies with human participants or human data. See also policy information about sex, gender (identity/presentation), and sexual orientation and race, ethnicity and racism.

Reporting on sex and gender	In this study, 6 males and 7 females were included in the healthy control group, and 5 males and 8 females were included in the MDD patient group. Both sex were grouped for analysis.
Reporting on race, ethnicity, or other socially relevant groupings	All participants are native Chinese people (Han ethnic group).
Population characteristics	The demographic information of human cohort was listed in Table S1. In brief, the healthy group had an average age of 27.0 years, and the patient group was 27.9 years on average.
Recruitment	The study sample was recruited based on the age (18~35 years), the sex ratio (approximately equal to the overall patient population admitted in the clinics), the diagnosis (DSM-5) and the absence of systemic disease or acute/chronic inflammation (which may confound the cytokine level). For the MDD group, the recruitment was performed by psychiatric clinicians from inpatients in the psychiatric ward. For the healthy control group, the recruitment was performed from the cohort in th routine body check of the same hospital. The possible bias in the recruitment may come from the subject selection bias in the clinician for recruiting. To reduce this bias, we have included a correlation analysis between the biological marker (LCN2) and clinical symptom (HAMA score). See Fig. 1c
Ethics oversight	Ethical approval for this study was obtained from the Research Ethics Board of Research Ethics Committee of the First Affiliated Hospital of Jinan University (Guangzhou, China). Informed consents have been obtained in written form from all participants before conducting the assay (see manuscript-Methods)

Note that full information on the approval of the study protocol must also be provided in the manuscript.

Field-specific reporting

Please select the one below that is the best fit for your research. If you are not sure, read the appropriate sections before making your selection.

☒ Life sciences ☐ Behavioural & social sciences ☐ Ecological, evolutionary & environmental sciences

For a reference copy of the document with all sections, see nature.com/documents/nr-reporting-summary-flat.pdf

Life sciences study design

All studies must disclose on these points even when the disclosure is negative.

Sample size	The sample size was determined based on literatures from the same field (e.g. Nature 2021:589, 426-430), and complied with animal welfare requirement and animal experimental ethical code.
Data exclusions	No data were excluded in this study.
Replication	All experiments were performed on multiple animals from different litters. The exact N number has been stated in the figure legend. For qPCR study or ELISA, triplicated biological samples were performed for each animal, and the averaged value was displayed.
Randomization	For all assays, mice were randomly assigned into each group before the initiation of experiments.
Blinding	All behavioral tests and in vivo calcium imaging (including data analysis) were performed by persons who were blinded to the group information. For other molecular and fluorescent imaging assays, at least 2 independent people were invited to analyze the same dataset, and the grouping information was blinded to people during data analysis.

Reporting for specific materials, systems and methods

We require information from authors about some types of materials, experimental systems and methods used in many studies. Here, indicate whether each material, system or method listed is relevant to your study. If you are not sure if a list item applies to your research, read the appropriate section before selecting a response.

Materials & experimental systems

n/a	Involved in the study	
☐	☒	Antibodies
☒	☐	Eukaryotic cell lines
☒	☐	Palaeontology and archaeology
☐	☒	Animals and other organisms
☒	☐	Clinical data
☒	☐	Dual use research of concern
☒	☐	Plants

Methods

n/a	Involved in the study	
☒	☐	ChIP-seq
☒	☐	Flow cytometry
☒	☐	MRI-based neuroimaging

Antibodies

Antibodies used

All antibodies used in this study were listed in Table S2.

Validation

Anti-LCN2 Antibody.RRID: PA5-20543. It was used to label the target protein LCN2 in PRL brain region; Ai19 mice, western blotting was applied at a concentration of 1:2000(https://www.thermofisher.cn/cn/zh/antibody/product/NGAL-Antibody-Polyclonal/PA5-79590)
Anti-SLC22A17 Antibody.RRID: PA5-79590. It was used as a marker of the target protein SLC22A17 in the PRL brain region; Ai19 mice, western blotting was employed at a concentration of 1:2000(https://www.thermofisher.cn/cn/zh/antibody/product/SLC22A17-Antibody-Polyclonal/PA5-20543)
Anti- c-Fos Antibody.RRID: 2250. It is used to stamp the immediate gene expression of model mice in PRL brain region; Ai19 mice, The concentration used in immunohistochemical experiment was 1:500(https://www.cellsignal.com/products/primary-antibodies/c-fos-9f6-rabbit-mab/2250)
Anti- TRPV1 Antibody.RRID: ab203103. Used to label pepper sensitive neuron TRPV1 in spinal cord; Ai19 mice, Its working concentration was 1:500(https://www.abcam.cn/trpv1-antibody-bs397-c-terminal-ab203103.html)

Animals and other research organisms

Policy information about studies involving animals; ARRIVE guidelines recommended for reporting animal research, and Sex and Gender in Research

Laboratory animals

Male C57BL/6J mice (5-6 weeks old) were purchased from Guangdong Medical Laboratory Animal Center. Animals were group-housed (4~5 per cage) in a air-conditioned room, with temperature maintained between 18 and 29 C, and the humidity between 40 and 70%, background noise below 60 dB.

Wild animals

No wild animals were used in this study.

Reporting on sex

Both male and female mice were tested. However, due to the absence of gender-specific effect of LCN2 (Fig. S1j-q), male mice were adopted in most of assays.

Field-collected samples

No field-collected samples were used in this study.

Ethics oversight

All animal experimental protocols have been pre-approved by the Ethics Committee of Experimental Animals of Jinan University in accordance with Institutional Animal Care and Use Committee guidelines for animal research.

Note that full information on the approval of the study protocol must also be provided in the manuscript.

Plants

Seed stocks

N/A

Novel plant genotypes

N/A

Authentication

N/A
